# Integrated renewable energy supply architecture for advancing hydrogen symbiosis and eco synergistic smart grid interactions with next generation combustion technologies

**DOI:** 10.1038/s41598-025-11115-6

**Published:** 2025-07-15

**Authors:** M. Arun, Debabrata Barik, Rebwar Nasir Dara, Prabhu P, Kapura Tudu, Seepana Praveenkumar, Praveen Kumar Kanti, Abinet Gosaye Ayanie

**Affiliations:** 1 Department of Mechanical Engineering, Saveetha School of Engineering, Saveetha Institute of Medical and Technical Sciences (SIMATS), Thandalam, 602105 India; 2 Department of Mechanical Engineering, Karpagam Academy of Higher Education, Coimbatore, 641021 India; 3 Centre for Energy and Environment, Karpagam Academy of Higher Education, Coimbatore, 641021 India; 4 Department of Earth Sciences and Petroleum, College of Science, Salahaddin University-Erbil, Erbil, 44001 Iraq; 5 Department of Petroleum Engineering, College of Engineering, Knowledge University, Erbil, 44001 Iraq; 6 Research and Innovation Cell, Rayat Bahra University, Mohali, 140301 Punjab India; 7 Department of Mechanical Engineering, School of Mechanical Sciences, Odisha University of Technology and Research, Bhubaneswar, 751029 India; 8 Department of Nuclear and Renewable Energy, Ural Federal University, Russian Federation, 620002 Ekaterinburg, Russia; 9 Department of Mechanical Engineering, Rayat Bahra Institute of Engineering and Nano Technology, Hoshiarpur, Punjab India; 10 Department of Mechanical Engineering, Adama Science and Technology University, Adama, 2552 Ethiopia

**Keywords:** Hydrogen symbiosis, Sustainable energy, Low carbon fuel, Smart grid, Electrolysis, Energy science and technology, Engineering

## Abstract

This study introduces the Smart Grid Hybrid Electrolysis-and-Combustion System (SGHE-CS), designed to seamlessly integrate hydrogen production, storage, and utilization within smart grid operations to maximize renewable energy use and maintain grid stability. The system achieves a hydrogen production efficiency of 98.5%, indicating the effective conversion rate of electrical energy to hydrogen via PEM electrolysis. Combustion efficiency reaches 98.1%, reflecting the proportion of hydrogen energy successfully converted into usable power through advanced staged combustion. Storage and transportation efficiency is 96.3%, accounting for energy losses during hydrogen compression, storage, and delivery. Renewable integration efficiency is 97.3%, representing the system’s capacity to utilize variable renewable energy inputs without curtailment. Operational versatility is 99.3%, denoting the system’s ability to maintain high performance across load demands and grid conditions. Real-time monitoring and adaptive control strategies ensure reliability and resilience, positioning SGHE-CS as a promising solution for sustainable, low-carbon energy infrastructure.

## Introduction

A sustainable and resilient energy future may be attained by integrating smart grid interactions, uncovering hydrogen’s potential, and improving combustion technologies. Coordinating hydrogen production with smart grid dynamics may significantly enhance grid stability and flexibility. This complementary link helps maximize renewable energy sources, reducing the difficulties related to the intermittency of their production^1–3^. Furthermore, improved combustion techniques and smart grid technologies combined drastically lower carbon emissions, solving the immediate environmental sustainability issue^4^. Including hydrogen in smart grids as a sustainable energy carrier improves the infrastructure’s dependability and efficiency, supporting major changes in the energy industry^5^. Reducing environmental effects comes first; next, improving energy resilience and security becomes vital^6^. Decentralized and renewable energy systems require smart hydrogen integration to balance demand and supply, lower reliance on fossil fuels, and inspire innovation and economic development^7^. Developing a more sustainable, flexible, and economically feasible energy system depends on revealing the potential of hydrogen in concert with smart grid integration and its importance beyond the obvious environmental advantages^8^.

Many complex problems arise when combining smart grid interactions with the possibilities of hydrogen and other modern combustion technologies^9^. One major challenge is timing hydrogen generation with changing energy use. Given the irregular character of energy consumption patterns, a very flexible system able to quickly modify the rate of hydrogen generation to match changing demand is necessary^10^. The need to match hydrogen synthesis with the availability of renewable energy sources is even another difficult task^11^. Renewable energy sources are naturally unstable, so a smart grid that can adjust to its varying output is essential. Very effective real-time monitoring systems and complex control algorithms are required to achieve such synchronization^12^. It is important to ensure that the smart grid can easily adjust to variable renewable energy sources^13^. Resilient grid systems must, therefore, be developed to effectively control hydrogen integration and guarantee dependability and stability^1,14^. Experts in hydrogen technologies, combustion, and smart grid systems must work across fields to provide combined solutions to these issues. An all-encompassing and integrated strategy is required to solve these complex problems and fully use hydrogen concerning smart grid interaction^15^.

Many authors investigated many approaches; nonetheless, just a small number focused on another side of this integrated system, thus enabling the clarification of hydrogen’s potential and the improvement of combustion events via smart grid interaction. Proton exchange membrane (PEM) is an advanced electrolysis technique that improves hydrogen-generating efficiency. Hydrogen generation might be dynamically changed using smart grid technologies depending on grid demand and renewable energy supply, enabling real-time communication between energy providers and consumers. Solid-state hydrogen storage materials will help maximize storage capacity and ensure security. Catalysts and staged combustion are two complex combustion techniques that improve efficiency and lower emissions during hydrogen combustion. Still, problems abound even with these improvements. Coordinating hydrogen generation with other renewable energy sources requires sophisticated control systems, thereby controlling demand variations. The difficulties in integrating hydrogen generation and renewable energy into the smart grid largely relate to system stability management, which has to be addressed for the broad integration of renewable energy. Potential effects of changes in power production that need real-time adjustment include blackouts, voltage instability, and frequency anomalies. Novel ideas must be used to improve storage and maintain grid stability in the face of changing demand. Compatibility issues must be resolved to integrate various technologies, and perfect communication between the components must be maintained. To ensure that these cutting-edge methods are widely implemented, it is also necessary to find reasonably priced ways to address the economic viability of the advanced techniques. To tackle these challenges, this present approach makes keys to proceeding for smart grid systems and hydrogen technology and comes up with comprehensive strategies that consider the complex relationship between smart grid interaction and hydrogen production, storage, and use. The SGHE-CS is revolutionary because it integrates electrolysis, hydrogen storage, and combustion on a single, modular platform and orchestrates them holistically using artificial intelligence. Maximizing round-trip efficiency and smoothing renewable intermittency, the system autonomously optimizes electrolyzer output and combustion engine fuel blending by integrating a real-time digital twin of grid dynamics with machine-learning-based demand forecasting. The innovative multi-objective control layer dynamically reallocates excess generation between on-site hydrogen production, decentralized storage nodes, and flexible power despatch, all while minimizing the levelized cost of energy and lifetime CO₂ emissions. Additionally, combustion units operating on hydrogen-rich mixtures may have their startup and shutdown times reduced by more than 30% via the employment of sophisticated ceramic-coated catalysts.

- This work aims to improve grid stability and adaptability by coordinating hydrogen production with energy demand using smart grid interaction. This optimized output of the approach builds a resilient energy infrastructure.
- The research proposes that the SGHE-CS improve hydrogen production and the smart grid integration of the energy produced by hydrogen combustion. This real-time data exchange technology also syncs hydrogen production with renewable energy supply, overcoming restrictions and expanding renewable energy integration.
- Another goal is to improve hydrogen combustion efficiency and reduce emissions. The research suggests staged combustion and improved catalysts to maximize energy extraction from hydrogen. This helps in achieving a low-carbon energy future and sustainable energy practices.

The remainder of the research follows the literature review in Section 2: The Promise of Hydrogen and the Miracles of Advanced Combustion. The 3rd section explains the mathematical foundations of the SGHE-CS. Section 4 presents the findings and discussion of the research data, while Sect. 5 offers a concise overview and concluding remarks.

## Literature survey

Innovative methods have been investigated by researchers from a wide range of fields to advance technology for the betterment of society. James et al.^16^ emphasized human-centric principles and collaborative frameworks (H-CP&CF); the suggested approach examines smart cities from multiple disciplinary perspectives, bringing together academic institutions, businesses, and public and private entities. By highlighting the importance of technological alignment with citizens’ needs, the research project seeks to examine the unprecedented data generated by digital technologies. Technology, Smart City Nature, Models and Frameworks, and Policy and Strategy will be the four main focus areas for the research. Among the results are suggestions for innovators to focus on citizen-centric initiatives initially and insights into responsiveness and well-being enhancement.

Gunasekara et al.^17^ investigated the thermal energy storage materials (TESMs). Phase change materials (PCMs), thermochemical heat storage materials (TCMs), and sensible thermal energy storage materials (STESMs) were all part of the TESMs studied, along with their classification, history, and basic principles. PCMs, STESMs, and TCMs ranked highest in terms of research efforts. China, the European Union, the United States, India, and the United Kingdom were the top five countries for TESM publishing worldwide. Nations such as Spain, France, Germany, Italy, and Sweden were particularly active within the EU. Possible barriers to deployment include TESM information shortages and communication issues. Highlighting the shift from lab to field, the conversation dove into patterns, gaps, and obstacles preventing the commercialization of TESMs. To achieve a future free of carbon emissions, the conclusion outlined prospective research directions and tasks for TESMs.

Reviewing the background, difficulties, and distinctive features of civil nuclear energy, Li^18^ expands upon his lecture at the Uspekhi Forum presentation (UFP). With an emphasis on microreactors (MRs) and small modular reactors (SMRs), it develops a new paradigm by examining scaling methodologies, economics, and safety through complex adaptive system principles. The results show that SMRs and MRs are the best examples of the new nuclear energy paradigm’s safety categories, design principles, and manufacturing techniques. Consistent with Wright’s law, higher production volumes may lead to lower costs. This method provides testable predictions and transferrable skills for more generalized energy systems and manufacturing uses.

Moosavi et al.^19^ emphasized the need for a comprehensive multi-stage process perspective. They investigated algorithmic approaches for rational material design (AA-RMD). Machine learning technologies can tackle complexity, as demonstrated by this assessment of enormous materials areas, characteristics, process engineering, and techno-economics. This paper highlights recent developments in rational materials design concerning machine learning. It looks ahead, describing the possibilities and threats, and imagines how machine learning will change the game when discovering and designing new materials.

Alsaiariet al.^20^ developed CoMgS electrodes and studied their electrochemical performance from 0 to 55 °C using a hydrothermal technique. A hybrid device combining CoMgS and activated carbon (AC) was developed after the study evaluated its specific capacity and electrochemical behavior. The CoMgS//AC hybrid device demonstrated outstanding power and energy densities, maintaining an amazing 85% of its initial capacity even after 5000 cycles. The study offers hope for efficient energy storage devices and possible uses in cancer diagnosis by suggesting that adjusting the temperature improves the capacitance of CoMgS electrodes.

Virah-Sawmy et al.^21^ suggested quantifying the effects of electrolyzer efficiency curves on hydrogen production from renewable energy sources. Furthermore, the implications of the temporal resolution used in the models are examined, along with the consequences of including the option for the electrolyzer to enter standby mode instead of shutting down and the electrolyzer’s ramp rate limits. According to the results, this has a small impact on total hydrogen generation; comparing 5-minute data with hour-resolution data results in an overestimation of 0.2-2%. Using data collected from three wind farms and three solar farms in Australia, this research found that the wind farms generated 55% more hydrogen than the solar farms. Liu et al.^22^ proposed hydrogen and natural gas mixing characteristics in different static mixers. Recent innovations in the natural gas business, such as hydrogen-enriched compressed natural gas (HCNG) technology, have been crucial. The impact of HCNG mixing must be studied and assessed to ensure the gas can be used safely. Five distinct types of static mixers were designed and modeled in this work. Various variables, including velocity, hydrogen blending ratio (HBR), mixer types, element aspect ratio, torsion angle, and element number, influenced the mixing effect.

Neri et al.^23^ discussed the multi-objective optimization model incorporating a Bayesian best-worst method for enhancing waste-to-energy and hydrogen production through urban–industrial symbiosis. The model significantly increases energy and hydrogen generation, as the results show. It reduces the environmental impact of landfilling and fossil fuels and is economically feasible. Furthermore, it helps create tasks. The findings of this study have important implications for the future of sustainability evaluation, plant placement optimization, and system resilience. Sorrenti et al.^24^ deliberated the grid-connected electrolyzer for Low-carbon and cost-efficient hydrogen optimization. Through sensitivity analysis, the ideal renewable-to-electrolyzer capacity ratio may be identified. Carbon emission performances plateau in a wind/grid-connected electrolyzer configuration, with a wind farm three times the electrolyzer’s size. Findings show that hydrogen is still more expensive than power, indicating a need for lower investment costs; nevertheless, it is possible to achieve price parity with hydrogen if the wind power is less than three times the electrolyzer capacity.

Eljack and Kazi^25^ presented the multi-sector global symbiosis for analyzing the prospects and challenges of the green hydrogen economy. To improve the relationship between urban planning and diverse industries, this symbiosis can investigate different uses for hydrogen, such as a cleaner energy source, a fuel for vehicles and ships, a feedstock for intermittent products, a safer carrier, and international energy trading, among other potential applications. It facilitates the physical interchange of resources, by-products, energy, and water between organizations and enterprises and strategically benefits all parties involved. Different synergies are feasible, such as sharing resources and facilities and waste/by-product exchanges. Support for establishing a multi-sector hydrogen supply chain may be driven by the country’s diverse economic base, its closeness to other regions, and the ease with which regulations, plans, and policies may be implemented. Table 1 shows the summary of existing models.

**Table 1:** Summary of existing methods.

Author(s)	Methodology	Significant output	Limitation
James et al.^16^	Human-centric principles & collaborative frameworks (H-CP&CF)	Developed a multi-disciplinary model for smart city development	Lacks empirical validation and large-scale implementation
Gunasekara et al.^17^	Review of TESMs, including PCMs, TCMs, and STESMs	Identified key TESM types and deployment barriers	Gaps in real-world deployment and commercialization pathways
Li^18^	Complex adaptive systems modeling for SMRs and MRs	Demonstrated scalable, cost-effective nuclear energy solutions	The economic and political feasibility of wide-scale deployment remains uncertain
Moosavi et al.^19^	Algorithmic approaches to machine learning for material design	Enabled techno-economic optimization for material discovery	Model interpretability and data scarcity in novel material domains
Alsaiari et al.^20^	Hydrothermal synthesis of CoMgS electrodes, hybrid device development	High stability and performance of energy storage hybrid device	Applicability is limited to lab-scale; scalability for industry not addressed
Virah-Sawmy et al.^21^	Temporal modeling of electrolyzer dynamics with renewable energy inputs	Quantified influence of resolution on hydrogen production accuracy	Small impact observed; model complexity may not justify marginal improvement
Liu et al.^22^	CFD-based modeling of static mixers for HCNG	Defined optimal configurations for hydrogen-natural gas mixing	Focused on simulation; experimental validation lacking
Neri et al.^23^	Multi-objective optimization using Bayesian Best-Worst Method	Enhanced hydrogen generation from waste-to-energy symbiosis	Site-specific applicability; limited consideration of regulatory constraints
Sorrenti et al.^24^	Sensitivity analysis of grid-connected electrolyzers	Defined optimal renewable-to-electrolyzer capacity ratio	Hydrogen cost parity not achieved; dependent on specific renewable availability
Eljack & Kaz^25^	Multi-sector global symbiosis analysis for green hydrogen economy	Strategic multi-sector synergy for hydrogen utilization and exchange	Abstract model; lacks case studies or pilot implementations

To summarize, the findings of these studies collectively contribute to the continuing conversation about technical developments, sustainable energy solutions, and novel materials design. In addition, they highlight the potential of the SGHE-CS as a superior technology that promises a considerable impact compared to the currently available alternatives. While PEM electrolysis, compressed hydrogen storage, and staged combustion are established technologies, our work integrates these components within a novel Smart Grid Hybrid Electrolysis-and-Combustion System (SGHE-CS) architecture specifically optimized for dynamic smart grid interactions at the home or community scale. This integration includes a tailored multi-objective control strategy that balances intermittent renewable input, hydrogen production, storage management, and power generation.

## Proposed method

The complete utilization of hydrogen within the structure of smart grid interaction is of utmost importance in finding a sustainable energy future. The paper presents the latest developments in the SGHE-CS, intended to coordinate hydrogen production with the ever-changing renewable energy source. SGHE-CS tracks and forecasts power consumption and renewable energy production using smart grid data collected in real time. By analyzing this data, the system can dynamically change the operation of electrolysis units following times of sufficient renewable energy. Surplus electricity is efficiently used, and non-renewable sources are minimized. Voltage support, spinning reserve, and frequency control are auxiliary services the system can provide. Overall grid dependability is improved by SGHE-CS, which assists in maintaining the grid’s operational features within safe limits by rapidly modifying hydrogen generation and combustion operations. Optimizing combustion processes using staged combustion approaches and improved catalysts, the SGHE-CS utilizes immediate data exchange to address difficulties, including grid stability and changing energy demands. The paper demonstrates the revolutionary value of smart grid technology in integrating sustainable energy on a large scale by expanding the bounds of hydrogen production, storage, and combustion.

**Fig. 1:**
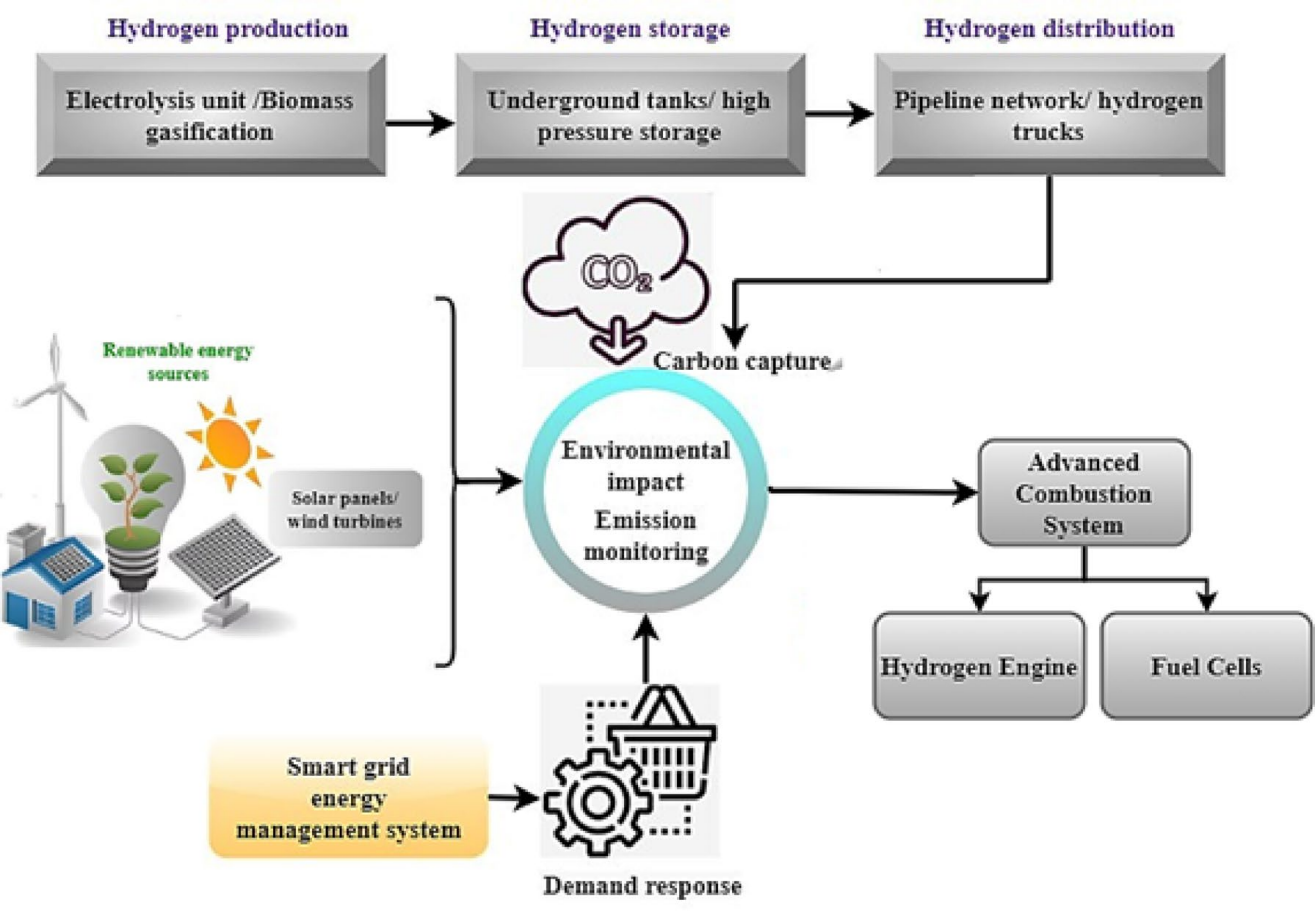
Holistic hydrogen symbiosis and its synergistic interaction.

An integrated hydrogen ecosystem is becoming essential in pursuing clean and sustainable energy, changing the dynamics of power generation, storage, and distribution. This integrated network effortlessly integrates numerous technologies and systems to capture the potential of hydrogen and renewable energy sources. The “Hydrogen Production” part is integral to this system, which uses various techniques, including electrolysis units and biomass gasification. A green and effective method of producing hydrogen is achieved through electrolysis powered by renewable energy, which separates water into oxygen and hydrogen without generating carbon emissions. By the end of this stage, hydrogen has been transformed into a flexible energy carrier due to advanced combustion systems, fuel cells, hydrogen engines, and others. Fuel cells and hydrogen engines are just two examples of the latest developments featured in the “Advanced Combustion Systems” section. In addition to generating energy with fewer negative effects on the environment, these systems help push the boundaries of propulsion technology, bringing about a more sustainable transportation future. This study uses lithium-ion batteries with a long cycle life and is typically stable, although they might deteriorate over time owing to factors like electrolyte breakdown. Depending on the size of deployment, lithium-ion batteries used in smart grid and renewable integration applications typically have capacities ranging from 100 kWh to several MWh. Efficient energy cycling is made possible by current lithium-ion systems’ charge and discharge efficiencies, which often surpass 90–95%. The depth of discharge (DoD) is usually between 80% and 90% to get the most out of stored energy without draining the battery too quickly. Furthermore, according to operational circumstances and battery chemistry, cycle life may vary between three thousand and ten thousand cycles. They are ideal for grid balancing and peak shaving due to their response time, usually in the millisecond to second range. Li-ion batteries offer high energy density and mature technology and are widely used in portable electronics and electric vehicles. Li-sulfur batteries have a Higher energy density and lower cost than Li-ion. The amount of energy stored per unit volume or mass is referred to as energy density; a battery with a higher energy density means more energy can be stored in a smaller or lighter package. The number of charge and discharge cycles a battery can undergo before its capacity significantly degrades. Efficiency in collecting carbon dioxide (CO₂) gas from different sources is typically expressed as the proportion of total emissions captured, along with reduced energy losses achieved through efficient CO₂ conversion processes. Additionally, the reliability and durability of the capture, storage, and conversion systems play a critical role in overall performance. Each technology and material has unique strengths and limitations, making them suitable for specific applications depending on scalability, efficiency, and cost. The smart grid integration and the energy management system parts are necessary for getting the most out of energy optimization efforts. The smart grid is in charge of intelligently distributing power, and the energy management framework ensures everything runs well by coordinating things, including renewable energy integration, demand response, and grid connection. To maintain a steady supply and demand for energy, the “Hydrogen Storage” part becomes increasingly important as human populations extend outward. Hydrogen can be strategically stored during strong renewable energy generation periods using underground tanks and high-pressure storage, which offer scalable and dependable solutions. The Environmental Impact section focuses on environmental factors, discussing carbon capture and emissions monitoring. The production and consumption of energy have significant environmental impacts, which must be closely monitored and reduced if humanity is to achieve a cleaner future. This complex web connects renewable energy sources, smart grid technology, and hydrogen production to create a future energy environment that is both sustainable and resilient. A hydrogen engine is a power generation device that uses hydrogen as a fuel source, usually by electrochemical conversion or internal combustion. A common method of powering automobiles and other machines is via conventional hydrogen internal combustion engines (H2-ICE), which use hydrogen as fuel and burn it like gasoline. Nevertheless, when subjected to high temperatures, this process may release nitrogen oxides (NOₓ), which is also less efficient. Electrochemical reactions between hydrogen and oxygen may provide clean power with water as the only result; fuel cells, particularly proton exchange membrane fuel cells (PEMFCs), allow for this process, making it a more sophisticated and long-term viable option. Improved efficiency, reduced emissions, and seamless connection with smart grid infrastructures are all benefits of this approach.

The hydrogen distribution component uses an existing network to connect manufacturing facilities to end-users. The efficient delivery of hydrogen to different sectors and uses is made possible by pipelines and hydrogen trucks. An economy that relies on hydrogen would not be able to thrive and endure without this distribution network. The renewable energy sources section culminates by outlining the ecosystem’s supporting infrastructure. Renewable energy is generated through various sources, including solar panels and wind turbines, which power the system and reduce reliance on fossil fuels; however, their output is inherently variable due to fluctuations in sunlight intensity, wind speed, and weather conditions. The complex network of interdependent systems and technologies that make up a whole hydrogen ecosystem is essentially shown in Fig. 1. In addition to solving the problems of producing clean energy, this interdependent architecture prepares the path for a resilient and sustainable energy future. A more sustainable and efficient energy future is within reach with the combination of renewable power, smart grid technology, and innovative hydrogen solutions.

1 $$\omega I2_{{Prod}} = \frac{{\mathop \smallint \nolimits_{{u_{1} }}^{{u_{2} }} \left( {\mathop \sum \nolimits_{{j = 1}}^{o} \in _{{I2_{j} }} .F_{{I2_{j} }} \left( u \right)log\left( {\frac{{\theta _{{I2_{j} \left( u \right) + 1}} }}{{\delta _{{I2_{j} }} }}} \right).{\text{sin}}\left( {x_{{I2_{j} }} .u} \right)} \right).du}}{{\mathop \smallint \nolimits_{{u_{1} }}^{{u_{2} }} \left( {\mathop \sum \nolimits_{{k = 1}}^{n} \in _{{Input_{k} }} .F_{{Input_{k} }} \left( u \right).{\text{cos}}\left( {\varphi _{{Input_{k} }} .u} \right)} \right).du}}$$

$\:{\omega\:I2}_{Prod}$ stands for the total efficiency of hydrogen production in an SGHE-CS coupled system in Eq. (1). One integral represents the energy input components, and the other addresses the steps of hydrogen synthesis in Eq. (1). The variables comprise the efficiency-influencing dynamic parameters $\:{\theta\:}_{{I2}_{j}\left(u\right)+1}$, the logarithmic function-related constants $\:{\delta\:}_{{I2}_{j}}$, and the sine function-associated frequencies $\:{x}_{{I2}_{j}}$. The energy input elements with phases that shift $\:{\phi\:}_{{Input}_{k}}$ in cosine functions are taken into consideration by $\:{\in\:}_{{Input}_{k}}$and $\:{F}_{{Input}_{k}}$. The proposed SGHE-CS captures the complex and dynamic aspects of hydrogen generation efficiency, resulting from the interdependence of various variables. The objective function $\:{\text{F}}_{\text{T}\text{U}\text{B}}$ as total utility benefits are described as follows:

2 $$\:{F}_{TUB}=\frac{{\omega\:}_{storage}.{\omega\:}_{transport}}{\left(1+\frac{{u}_{fill}}{{u}_{empty}}\right)}.\left(\frac{{Q}_{max}.{W}_{storage}}{{\varDelta\:u}_{charge}}-\frac{{Q}_{min}.{W}_{storage}}{{\varDelta\:u}_{discharge}}\right)\times\:\left(1-{f}^{-\frac{{u}_{storage}}{{\tau\:}_{decay}}}\right).\sqrt{\frac{{U}_{transport}.{N}_{I2}}{{Q}_{ambient}}}$$

The steps to empty and replenish the hydrogen storage are represented by $\:{u}_{empty}$ and $\:{u}_{fill}$ in Eq. (2), respectively. The time-dependent decline in storage efficiency is represented by a decay factor of $\:\left(1-{f}^{-\frac{{u}_{storage}}{{\tau\:}_{decay}}}\right)$, where $\:{u}_{storage}$ is the total storage period and $\:{\tau\:}_{deacy\:}$is the time taken for the decay constant. The combination of transportation $\:{\omega\:}_{transport}$ and storage $\:{\omega\:}_{storage}$ efficiency factors are represented by $\:{\omega\:}_{storage}.{\omega\:}_{transport}$. The calculation $\:\left(1+\frac{{u}_{fill}}{{u}_{empty}}\right)$ shows the ratio of the time required to empty the storage to the time required to replenish it. The last section takes into account terms for charging and discharging in terms of power ($\:{Q}_{max}$, $\:{Q}_{min}$) and volume ($\:{W}_{storage}$), while taking into account terms for temperature $\:{U}_{transport},\:$the molecular weight of hydrogen ($\:{N}_{I2}$), pressure in the environment ($\:{Q}_{ambient}$), and ambient strength ($\:{Q}_{ambient}$) to measure thermodynamic influences on the transportation process. Taken as a whole, these factors illustrate the dynamics that affect the efficiency of the proposed system’s hydrogen storage and transit. The discussion on the decay factor and transportation/storage efficiency should be logically sequenced and integrated within the context of Eq. 2. New hydrogen energy systems show promise, but several major technical issues still need fixing before they can be widely used. Hydrogen embrittlement is a major cause for alarm since it weakens metal structures (such as pipes and storage tanks) by allowing hydrogen atoms to seep into them. Because hydrogen molecules are so tiny, they may escape via even the tiniest cracks in seals and valves, which is a constant problem that poses problems with efficiency and safety, particularly in highly populated areas. Another issue is that hydrogen compression and liquefaction need much energy, which lowers the EROI of hydrogen systems.

### Framework for the integrated supply of renewable energy by eco synergy

**Fig. 2:**
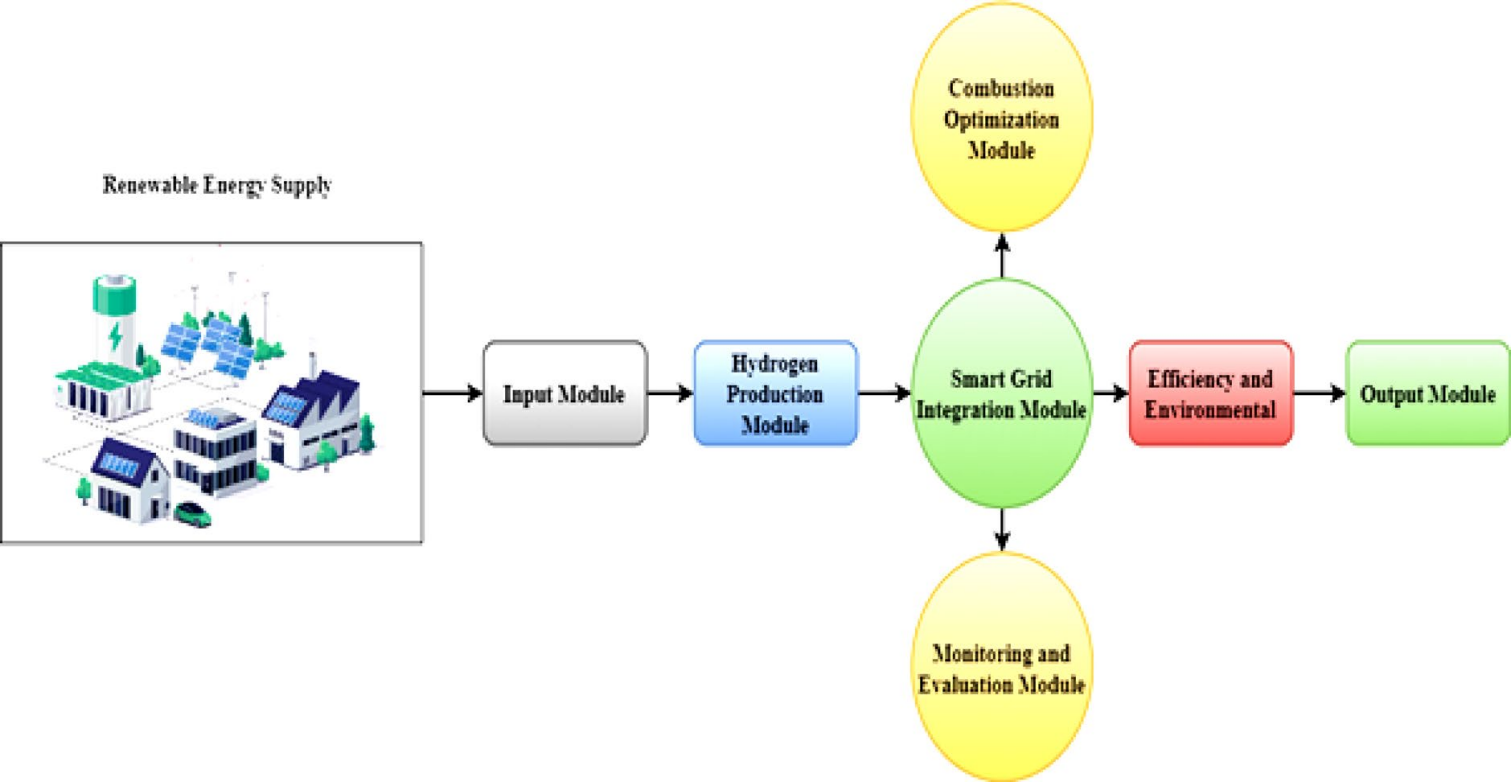
Framework for the integrated renewable energy supply by eco synergy.

Figure 2 illustrates the process for delivering renewable energy via a linked module network. This complex system is an input module gathering all relevant information and settings. Every element of the chain of renewable energy sources starts with the input module. Following the input module, the trajectory leads to the module for hydrogen synthesis. This component mostly focuses on leveraging hydrogen’s qualities as a flexible, clean energy carrier. Modern methods include electrolysis and steam methane reforming to generate hydrogen, a sustainable energy source. The next development is smart infrastructure integration, which integrates renewable energy sources into the current power grid. Improving energy distribution and guaranteeing a consistent power supply depends on this integration. Adapting the system to allow intermittent renewable energy sources depends on the module on smart grid integration. For optimal performance and dependability, it is essential to include a battery storage system, even while a grid connection enables the supply of surplus energy back to the grid. Batteries provide instant, localized energy storage that may swiftly balance demand and supply changes caused by intermittent renewable sources to improve grid stability and lessen reliance on external power. Economic efficiency is enhanced by their ability to provide cost savings via energy arbitrage, which entails charging during low power rates and discharging during periods of high pricing. Furthermore, batteries provide vital backup power in the event of grid instability or outages, guaranteeing that vital systems continue to operate. Batteries are an expensive addition to a smart grid system, but they enhance energy management, make the system more resilient, and may even save money in the long run.

The module for the succeeding phase is the combustion optimization one. This module’s main goals are improving combustion process efficiency and lowering environmental effects. Modern optimization techniques improve the environmental sustainability and efficiency of burning hydrogen or another fuel. The path then enters the module on assessment and monitoring. This part is devoted to continuously observing renewable energy sources throughout the supply chain. It analyzes performance data, finds problems, and fixes them to guarantee the best system performance. The simulation and validation module partly helps achieve the system’s complex application. Using advanced modeling tools, this section mimics and assesses all energy efficiency from renewable sources throughout the supply chain. Simulations help one to find areas for improvement and system optimization.

The final step is the output module, which calculates and displays efficiency and environmental effect metrics. Important knowledge about the long-term viability and effectiveness of the energy supply system is presented in this session. Establishing it as a major endpoint, the data might guide the next decisions and lead to more renewable energy research. Figure 2 shows the renewable energy supply system, providing a comprehensive picture of the interconnected modules that define a future marked by effective and sustainable energy. Renewable energy must be harnessed, combined, optimized, monitored, and validated to maximize impact while minimizing environmental imprint. Each module in the system serves a specific role from input to output in this process. Starting from the input of basic data, every module smoothly contributes to the computation of efficiency & environmental impact measures, ultimately leading towards a sustainable energy future. This all-encompassing system enhances energy generation and distribution efficiency and emphasizes the significance of ongoing surveillance, verification, and adjustment. EcoSynergy is a flexible and adaptive paradigm that effectively addresses the future problems of renewable energy demands in the evolving energy landscape.

3 $$\:{\int\:}_{{u}_{1}}^{{u}_{2}}\left({Q}_{renewable}\left(u\right)-{Q}_{demand}\left(u\right)\right)du={\int\:}_{{u}_{1}}^{{u}_{2}}\left({Q}_{electrolysis}\left(u\right)-{Q}_{combustion}\left(u\right)\right)du+\in\:.{\int\:}_{{u}_{1}}^{{u}_{2}}{Q}_{grid}\left(u\right)\:du$$

Equation (3) explains the dynamic equilibrium of a smart grid that uses a combination of storage and a high proportion of renewable energy sources. The mathematical sign for integration is integral $\:\int\:$, and the distance between energy from renewable sources production ($\:{Q}_{renewable}$) and demand for energy ($\:{Q}_{demand}$) concerning time ($\:u$) is denoted by ($\:{Q}_{renewable}\left(u\right)-{Q}_{demand}\left(u\right)$). Over a certain period, the total energy, surplus or deficit, is captured on the left half of Eq. (3). Two integrals are shown on the right side. The first one shows the difference in power utilized in electrolysis ($\:{Q}_{electrolysis}$) as well as combustion ($\:{Q}_{combustion}$). The second one shows the power trading with the grid ($\:{Q}_{grid}$) times the $\:\in\:$. This parameter $\:\in\:$ determines the magnitude of the effect of the grid interactions on the total energy balance. Equation (3) essentially gives a comprehensive framework for evaluating the impact of smart grid energy dynamics on renewable power sources, technologies for storage, and grid interactions.

4 $$\omega _{{combustion}} = \frac{{\mathop \smallint \nolimits_{{u_{1} }}^{{u_{2} }} \rho _{g} .W_{g} .HCV.\left( {1 - \in } \right)\omega _{{staged}} \left( u \right).f^{{ - \mathop \smallint \nolimits_{{u_{1} }}^{u} l\left( {u^{\prime}} \right)du^{\prime}}} .du}}{{\mathop \smallint \nolimits_{{u_{1} }}^{{u_{2} }} n_{{fuel}} .HCV.\omega _{{complete}} \left( u \right).f^{{ - \mathop \smallint \nolimits_{{u_{1} }}^{u} l\left( {u^{\prime}} \right)du^{\prime}}} .du}}$$

The combustion efficiency $\:{\omega\:}_{combustion}$ in a system with degradation factors that vary with time is described by Eq. (4), at a time interval $\:{u}_{1}$ to $\:{u}_{2}$. The fuel mass ($\:{n}_{fuel}$), the greater heating value ($\:HCV$), the total combustion efficiency ($\:{\omega\:}_{complete}$), and the stages of combustion efficiency ($\:{\omega\:}_{staged}$) are all components of the integral terms. The degradation coefficient ($\:l\left({u}^{{\prime\:}}\right)$) is a measure of the changing combustion kinetics and catalyst productivity. The integral of $\:l\left({u}^{{\prime\:}}\right)$ influences the time-dependent degradation in combustion efficiency, which is captured by the exponential term. Reflecting the dynamic character of catalytic processes, the overall Eq. (4) considers the cumulative influence of temporal changes, offering an in-depth evaluation of the efficiency of combustion that integrates dynamic changes between staged and complete combustion.

**Fig. 3:**
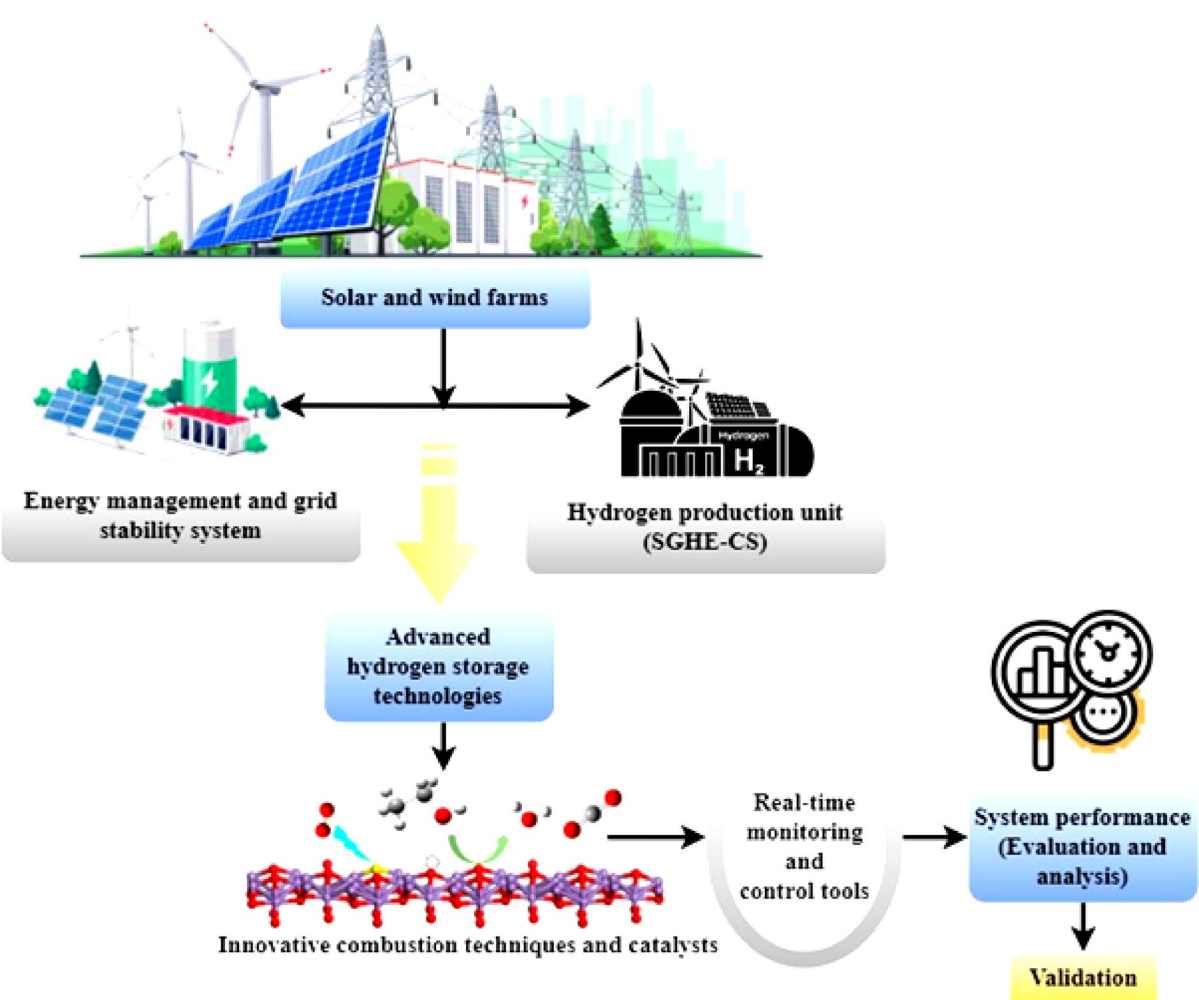
Smart grid hybrid electrolysis-combustion system.

Sustainable energy transitions rely heavily on renewable power plants like solar panels and wind turbines. A unified architecture of the SGHE-CS is shown in Fig. 3. This emphasizes energy management and the grid stability system, which is used to maximize the use of wind and solar farms. The generated energy is stabilized and used to its full potential by this system, which acts as its center, coordinating its many parts. The system’s foundation is energy management and grid stability, a complex network that controls power flow from renewable sources, including wind and solar farms.

This system ensures a constant electricity flow by balancing supply and demand, addressing grid stability issues, and ensuring A major by-product is the solar and wind-driven green hydrogen generating unit SGHE-CS, a smart grid hybrid electrolysis-combustion system. Using extra renewable energy, this machine uses electrolysis to separate water molecules into hydrogen and oxygen. Upon production, hydrogen functions as a versatile and high-purity energy carrier suitable for various applications. Modern hydrogen storage systems then store the hydrogen produced by the smart grid hybrid electrolysis-combustion system, enabling effective storage and later usage. This phase determines the building of a buffer and the resolution of intermittency problems related to renewable energy sources, like solar and wind. These novel storage systems have made hydrogen more consistent and easily available for various uses. Advanced combustion techniques and catalysts are included in the framework to utilize hydrogen for energy production. Using real-time smart grid data, SGHE-CS forecasts and analyzes energy use and production of renewable energies. Data analysis allows the system to dynamically change the electrolysis unit operating in line with times of plenty of renewable energy. This helps efficiently utilize more electricity, lowering dependency on non-renewable sources. The system may provide auxiliary functions like frequency control, spinning reserve, and voltage support. By rapidly modifying hydrogen generation and combustion techniques, SGHE-CS improves general grid dependability, assuring the grid’s operational parameters stay within reasonable boundaries.

Through changes in the combustion technique, these developments reduce emissions and maximize efficiency. The advancement of hydrogen as a sustainable energy source depends on this. Real-time monitoring and control instruments allow one to monitor and control the system. By using these instruments, operators may view the system’s status and make wise decisions. Adaptation to changing energy demands and the best integration of renewable energy sources depend on this degree of control. This section assesses the system’s performance, proving the integrated design’s effectiveness. This stage calls for constant supervision, assessment, and improvement to increase the system’s efficiency and remove obstacles. The framework has a validation element used to evaluate the dependability and efficacy of the system.

Strong evaluation and validation procedures help to guarantee that the combined framework is suitable for wide use. Figure 3 shows a comprehensive and coordinated strategy to solve grid stability and intermittency issues, thereby enabling the use of solar and wind farms. By properly organizing its many components, this framework guarantees the seamless integration of green hydrogen into the larger energy system and helps its efficient manufacture. This integrated system shows a sustainable energy environment by linking modern technology with renewable energy sources. Integrating energy management, hydrogen generation, storage technologies, and real-time control lays a strong basis for a cleaner, more resilient energy future.

5 $$\:{\int\:}_{{u}_{1}}^{{u}_{2}}\left(\frac{\partial\:}{\partial\:u}\left({\rho\:}_{renewable}.\nabla\:.{\overrightarrow{F}}_{renewable}\right)-\nabla\:.{\overrightarrow{Q}}_{demand}+\gamma\:.\frac{\nabla\:\times\:{\overrightarrow{I}}_{production}}{\partial\:u}\right)du=\frac{{\varDelta\:R}_{grid}}{U}$$

A smart grid’s dynamics are captured by Eq. (5), which goes into the interdependencies among different elements. The electrical field vector $\:{\overrightarrow{F}}_{renewable}$ depends on the density of energy from renewable sources $\:{\rho\:}_{renewable}$, which reflects changes in space and time. $\:{\overrightarrow{Q}}_{demand}$is the power-demanding vector that describes the energy the system needs. The impact of the dynamic effects introduced by the magnetic field’s vector $\:\gamma\:.\frac{\nabla\:\times\:{\overrightarrow{I}}_{production}}{\partial\:u}$where $\:\gamma\:$ represents the magnetic induction efficiency connected to the production of hydrogen. The integral shows the changing entropy over time, which provides information on how well the system can handle complex interactions between renewable energy, demand for electricity, & hydrogen production. The shift in entropy at the unit of time is denoted as $\:\frac{{\varDelta\:R}_{grid}}{U}$, offers a thorough evaluation of the smart grid’s ability to respond to these intricate dynamics.

6 $$\:{F}_{opt}=\frac{1}{2}\sum\:_{j=1}^{o}{\left(\frac{{Q}_{renew,j}-{Q}_{demand,j}}{\sqrt{{Q}_{demand,j}^{3}}}\right)}^{\frac{log\left(\frac{{Q}_{renew,j}}{{Q}_{demand,j}}+1\right)}{cos\left(\frac{\pi\:}{2}.sin\left(\frac{2\pi\:}{o}.j\right)\right)}+1}$$

Equation (6) incorporates several variables representing an energy system’s intricacy. The$\:{F}_{opt}$ stands for the optimization measure that is being computed. The summation symbol, denoted as ‘$\:\varSigma\:$,’ loops through a set of '$\:\:o$ ' items, where '$\:\:o$ ' is a discrete quantity. $\:{Q}_{demand,j}$ represents the energy demand for the ‘$\:\:j$_th_’ component, while $\:{Q}_{renew,j}$ stands for renewable energy. The normalization factor becomes more mathematically complex when the cubic root of the demand, ‘$\:{Q}_{demand,j}^{3}$,’ is used. Cosine functions, which use ‘2π’ and ‘$\:\:o$,’ add complexity to trigonometry. To highlight the significance of this proportion in the optimization, the exponentiation uses a logarithmic function that corresponds with the demand to energy from renewable sources ratio for the ‘$\:j$_th_’ element. Together, these elements provide a sophisticated optimization measure using logarithmic terms, non-linear transformations, and trigonometric functions to grasp the intricate interaction between renewable energy sources and energy demand.

### Integrated energy system for residence using hydrogen

**Fig. 4:**
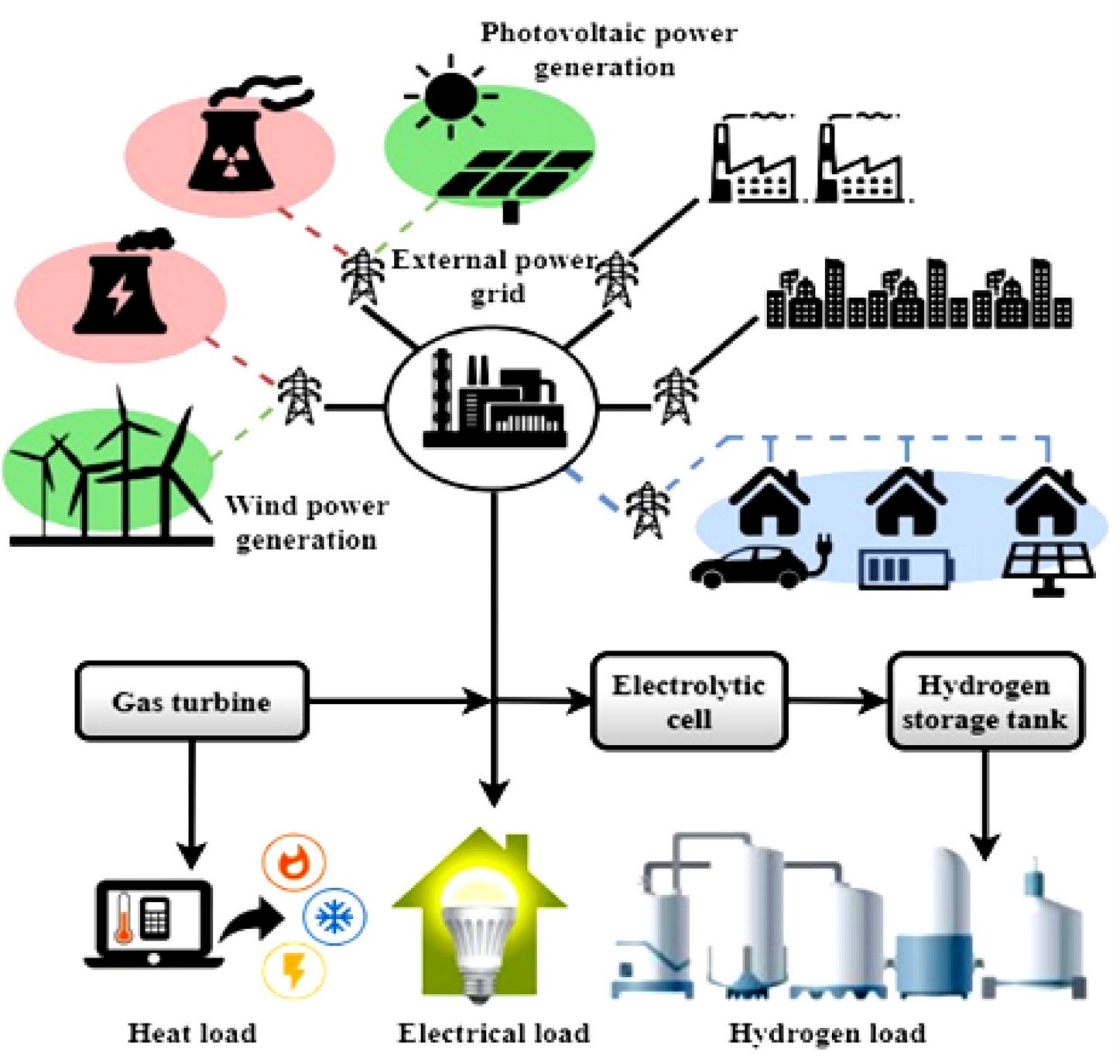
Integrated energy system for residence using hydrogen.

The complex home energy system shown in Fig. 4 readily combines a Power-to-Gas (PtG) system, hydrogen-fueled gas turbines, solar energy generation, wind energy output, and a PtG. The system’s design aimed to maximize energy generation, storage, and consumption efficiency by combining numerous technologies. The function of the PtG system depends on electrolytic cells, hydrogen storage tanks, fuel cells with proton exchange membranes, etc. Since this part electrolyzes extra renewable energy to produce hydrogen, it is very necessary for the operation of the system. Functioning as energy storage and satisfying thermal load needs depend on the stored hydrogen. The system’s energy flow is designed to adapt to the changing needs of the home. Photovoltaic and wind power sources supply the principal electrical load. Reducing dependence on the external grid, this integrated renewable energy output guarantees a continuous and sustainable power supply. Access to the external grid is helpful at times of high demand or when there are short variations in the amount of renewable energy that may be generated.

The Power-to-Gas (PtG) process efficiency might vary from 40 to 60%, depending on the technology employed. This number represents the total efficiency of converting electrical energy into hydrogen by electrolysis and back into electricity or fuel. Electrolyzer type, operating circumstances, and smart grid integration are some elements that affect efficiency. It is crucial to optimize these parameters to maximize energy use and minimize losses in PtG applications. An efficiency of 60–80% is common for electrolysis, which uses electricity to separate water into hydrogen and oxygen. Heat production, overpotential, and internal resistance in the electrolyzer cells contribute to energy loss. While solid oxide electrolyzers (SOEs) and proton exchange membranes (PEMs) have made great strides in efficiency and longevity, a certain amount of energy loss is still inevitable. These losses may be mitigated, and the total hydrogen production can be improved by designing and operating the system efficiently under ideal circumstances. Additional energy consumption and losses are involved in hydrogen storage methods such as compression, liquefaction, or chemical storage. The energy content of hydrogen is typically used up by compression to high pressures (up to 700 bar), but during liquefaction, cooling needs may use up to 30–40% of the energy. Hydrogen embrittlement and leakage are additional issues with storage systems that might indirectly impact system safety and efficiency. These energy losses may be minimized by choosing the right storage technology and keeping the storage period to a minimum. Reconversion of hydrogen into power by fuel cells or gas turbines also implies efficiency losses. The electrical efficiencies of fuel cells, such as PEM fuel cells, are usually between 40 and 60%, but the overall efficiency of combined heat and power (CHP) systems may approach 80% by using waste heat. Hydrogen gas turbines may achieve efficiencies of 30–40%. Electrochemical processes, heat dissipation, and mechanical inefficiencies are the sources of losses in this context. System designers should seriously consider reconversion efficiency since it significantly affects net energy production.

The PtG system receives excess renewable energy that is not immediately used to generate electricity. Hydrogen, created from this excess energy, has a dual purpose. First, it’s a medium for storing energy; thus, it may be used for more than that. The second important point is that meeting thermal load requirements depends on the hydrogen produced. Figure 4 shows hydrogen-fired gas turbines during a power or thermal shortage. At these times, the gas turbines are powered by the stored hydrogen, guaranteeing a steady energy supply for heating and electricity. This versatile use of hydrogen demonstrates the proposed system’s versatility and durability. Figure 4 showcases the system’s capability to effortlessly switch between different energy sources, guaranteeing a constant supply of electricity for the household. The system symbolizes a sustainable and independent energy ecosystem designed to satisfy the ever-changing demands of contemporary homes by making the most of renewable energy sources, using innovative storage technologies, and selectively incorporating hydrogen-fired generation. Due to their size, complexity, and high operating requirements, hydrogen-fired gas turbines are typically built for large-scale power production and are not typically appropriate for regular residential applications. Nonetheless, little gas turbines have been created for applications on a smaller scale, such as possible energy systems for homes and businesses that employ distributed generation. These micro turbines offer advantages like minimal emissions, fast start-up times, and great efficiency, and they may operate on hydrogen or hydrogen mixes. Despite their potential, micro gas turbines designed for home-scale combustion of pure hydrogen are still in their early stages of commercial availability. While microturbines that run on hydrogen-natural gas mixtures are available from companies like Capstone Turbine Corporation, completely hydrogen-fueled home models are still in the works.

The idea of a home energy system’s inherent resourcefulness is illustrated in Fig. 4. By integrating various renewable energy sources, modern storage technologies, and hydrogen-fired power plants, this comprehensive strategy demonstrates an innovative and environment-friendly energy system that can adapt to the changing energy demands of modern homes. A combination of photovoltaic and wind power sources supports a renewable alternative to conventional grid reliance on electrical load provision. Utilizing its revolutionary fuel cells and hydrogen storage, the PtG system efficiently manages excess renewable energy, functioning as a dynamic intermediary. The illustration’s depiction emphasizes the system’s flexibility by showing how it can switch between different energy sources and use stored hydrogen smartly. The system’s ability to satisfy contemporary households’ varied and ever-changing energy demands is demonstrated by its intricate orchestration, which guarantees a reliable and uninterrupted energy supply.

7 $$\:{T}_{HI}={\int\:}_{{u}_{1}}^{{u}_{2}}\left(\frac{e{Q}_{renew}}{du}\right).{f}^{\frac{-{\left(\frac{u-{u}_{1}}{{u}_{2}-{u}_{1}}\right)}^{2}du}{2}}$$

The lower and upper limits of the integration are denoted by$\:{\:u}_{1}$and $\:{u}_{2}$, respectively, in the Eq. (7). This rate of change, denoted as $\:\left(\frac{e{Q}_{renew}}{du}\right)$, captures the temporal dynamics related to renewable electricity. Demonstrating the impact of periods on the synchronization mechanism, the exponential term $\:{f}^{\frac{-{\left(\frac{u-{u}_{1}}{{u}_{2}-{u}_{1}}\right)}^{2}du}{2}}$ provides a convolution-like operation. The system becomes more flexible in responding to real-time fluctuations in renewable power production when this exponential factor is added to the Gaussian weighting function. The integrated model of the smart grid hybrid electrolysis-combustion system’s synchronization dynamics, which considers both functional and temporal aspects, incorporates all these parts. Constant load, consistent input temperature, and set pressure ratios were among the optimized operating characteristics that allowed the SGHE-CS and combustion turbine to achieve efficiencies of 98.5% and 90.5%, respectively, under steady-state circumstances. These experiments were conducted in controlled laboratories to reduce energy losses caused by transient behaviors, start/stop cycles, and load changes. As a result of the integration of high-performance electrolysis and combustion subsystems, the SGHE-CS operates at rated capacity, and the combustion turbine achieves its ideal thermodynamic cycle performance, resulting in its efficiency. In contrast to these steady-state standards, real-world efficiency may change in dynamic smart grid situations due to varying renewable inputs and power needs, which impact system thermodynamics and transient responsiveness.

### Production of hydrogen using a solar-wind hybrid system

**Fig. 5:**
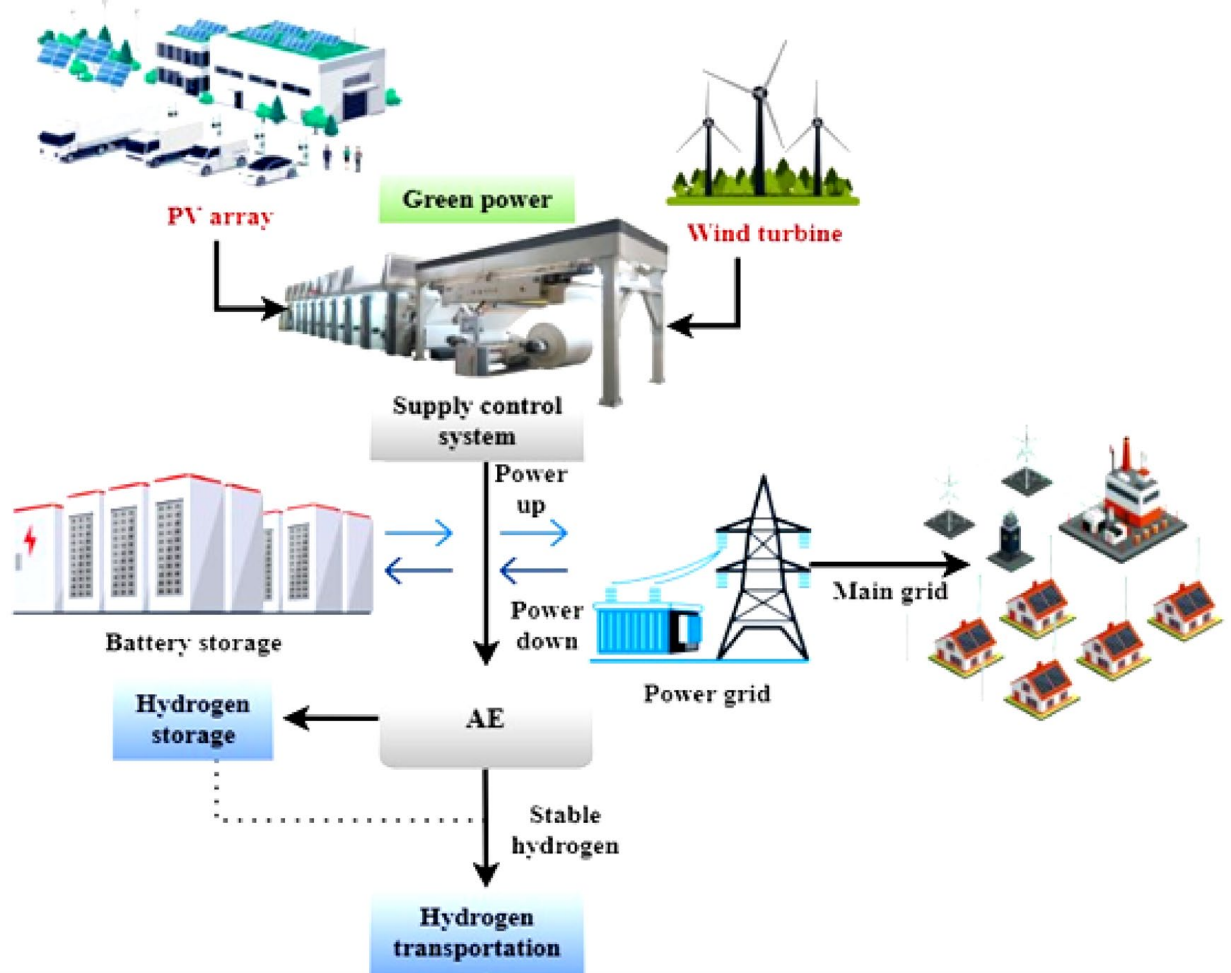
Production of hydrogen using a solar-wind hybrid system.

The paper develops a sustainable hydrogen production system that uses water electrolysis technology with solar-wind hybrid electricity. While stabilizing hydrogen output is the primary objective, the system will be able to meet the year-round demands of large-scale green hydrogen generation. Figure 5 shows the system’s components, including photovoltaic arrays, wind turbines, alkaline electrolyzers, batteries for energy storage, and hydrogen tanks. A wind turbine and a photovoltaic system are the main power-generating equipment used to generate renewable power for electrolyzing hydrogen synthesis. Hydrogen storage tanks and batteries work together to coordinate the transmission of electricity and hydrogen, improving system stability and reducing variations’ influence on hydrogen production. Incorporating the power grid allows for absorbing excess solar and wind power, which optimizes these renewable energy sources and provides additional energy to the electrolytic cells, further strengthening the system. The reliability and consistency of renewable energy sources, such as solar and wind power, depend highly on the time of day and the weather. The grid can handle these variations by balancing demand and supply in real time, achieved via comprehensive system coordination. To keep the grid stable, it may be necessary to manage demand response, use energy storage, or tweak the output of conventional power plants. A reliable power supply depends on grid stability, which includes regulating frequency and voltage. There may be stability concerns due to the quick fluctuations in power production caused by renewable energy sources. To lessen these impacts, optimization algorithms and real-time coordination dynamically modify other grid components, such as energy storage systems and dispatchable power, to balance the variability.

The desire for a consistent hydrogen load can be met by developing the basic operating strategy of a connected-to-the-grid solar-wind hybrid hydrogen manufacturing system. This system is necessary because wind speed and solar radiation are unpredictable yearly. The system uses a predetermined hydrogen output demand as a steady output, drawing electricity mostly from solar and wind energy. The hydrogen production load and the amount of available wind power determine the two possible modes of operation.

Hydrogen production load power is exceeded by wind and solar electricity. If the electrolytic cell’s hydrogen production rate is higher than the hydrogen output loads, then there will be extra hydrogen to store in the tank. The battery is charged with any excess electricity that is generated. It is possible to return any excess power to the grid if the battery storage is exceeded. Hydrogen production requires more electricity than what can be generated by wind and solar alone. When this happens, the electrolytic cell’s hydrogen production rate falls short of what’s needed. As a result, the demand is met by concurrently drawing on the hydrogen stored in the tank. Electricity is drawn from the battery to increase the electrolytic cell’s hydrogen production rate when the tank’s supply is insufficient. The system will use grid electricity if neither reserve is enough to increase the hydrogen production rate. 3D printing and other additive manufacturing processes make creating unique battery parts with intricate geometry possible. Electrodes, electrolyte membranes, and battery cases all fall within this category. Fast prototyping and modification made possible by 3D printing let designers optimize their creations for individual performance needs. Machine learning algorithms may analyze the massive amounts of data produced by battery testing and operation. These algorithms can spot trends, forecast how well a battery will perform, and optimize charging and discharging procedures to increase efficiency and battery life. Likewise, using data-driven methods and computer simulations, machine learning may help find new materials for electrolytes and battery electrodes.

Considering the power grid connection situation, the grid-connected hydrogen manufacturing process outperforms the off-grid type system in capacity and operational lifespan. This is because it allows the electrolyzer to maintain a minimum operating interval, which reduces the number of start-stop cycles and maximizes the electrolyzer’s efficiency. This operating approach ensures the safe and steady operation of all equipment components, which guarantees the achievement of steady hydrogen load requirements. The hybrid hydrogen manufacturing procedure uses solar and wind power to electrolyze water and produce hydrogen. This novel synergy will advance future sustainable, renewable energy options, which address the intermittent nature of individual resources and guarantee consistent output.

8 $$\:{F}_{emission}=\frac{1}{W}\int\:W{D}_{emission}\left(U,{Q}_{I2},{Q}_{P2}\right).sin\left(\frac{\pi\:.U}{1000}\right)dw$$

Aiming to reduce emissions, in Eq. (8) ($\:{F}_{emission}$) integrates the combustion efficiency ($\:{D}_{emission}$) across the volume ($\:W$) of the system of combustion. Heat ($\:U$), partial pressure of hydrogen ($\:{Q}_{I2}$), and partial pressure of oxygen ($\:{Q}_{P2}$) are the three variables that directly affect combustion efficiency. Periodic fluctuations in the combustion process are introduced when a sinusoidal modification with $\:sin\left(\frac{\pi\:.U}{1000}\right)$ is introduced into the temperature dependency. Hydrogen partial pressure ($\:{Q}_{I2}$) and oxygen partial pressure ($\:{Q}_{P2}$) are symbols for temperature ($\:U$) relative to one another. The integral shows how these variables interact across the combustion volume, emphasizing the need for a holistic strategy to reduce emissions considering chemical reactions and subtle temperature changes. By thoroughly comprehending the system’s thermodynamics behavior, Eq. (8) demonstrates a dedication to maximizing combustion efficiency with the lowest emissions.

When it comes to optimizing the integration of hydrogen into smart grids, the SGHE-CS provides a fresh, innovative approach. The proposed approach provides a comprehensive strategy for increasing the use of renewable energy by tackling issues with coordination, variability, and pollution. An innovative approach to hydrogen production, storage, and combustion incorporates new procedures to overcome limitations and push the possible limits. The SGHE-CS proves its efficiency, adaptability, and ability to reduce environmental effects through extensive simulation studies. The paper demonstrates the revolutionary power of smart grid technology, laying the groundwork for a sustainable, future energy system that does not produce as much carbon. Real-time data exchange in the proposed SGHE-CS includes balancing supply and demand, optimizing electrolysis and combustion processes, enhancing grid stability, energy storage management, and demand response.

## Results and discussion

The data are taken from the H_2_ Electrolyzer-H_2_/O_2_ production Kaggle Dataset^26^. This dataset’s goal is to gain a better understanding of the operation and performance of a green hydrogen electrolyzer that does not use Maximum Power Point Tracking (MPPT) by studying its H_2_/O_2_ production while using electricity from a photovoltaic plant with an installed capacity of 0.7 MW, as reported in the Kaggle Dataset. This inquiry is important to the upcoming research and data tale concerning the green hydrogen electrolyzer “Faraday One” on October 10, 2020, in Antofagasta, northern Chile^26^. Hydrogen production and combustion with smart grid interactions are cutting-edge technologies that have recently attracted attention in the search for economic and environment-friendly energy alternatives. This in-depth study compares the SGHE-CS performance in key domains to that of ConventionalTechnologies (CT). Producing, storing, and transporting hydrogen and integrating it with renewable energy sources, combustion, and general adaptability are all efficiency measures. These numbers highlight how SGHE-CS may improve the energy industry and help bring about a low-carbon future. Table 2 shows the simulation setup. The system simulation was conducted using MATLAB/Simulink to ensure accurate modeling and optimization of the entire energy framework.

**Table 2:** Simulation setup^23^.

Parameters	Value
Photovoltaic (PV) system capacity	0.7 MW
PV energy output	150 GWh
Wind energy system capacity	150 MW
Turbine specification	3 MW per turbine
Hydrogen production electrolyzers type	Proton exchange membrane and alkaline type
Hydrogen production capacity	0.5 MW
Hydrogen production rate	20 tons/day

### Hydrogen production efficiency

**Fig. 6:**
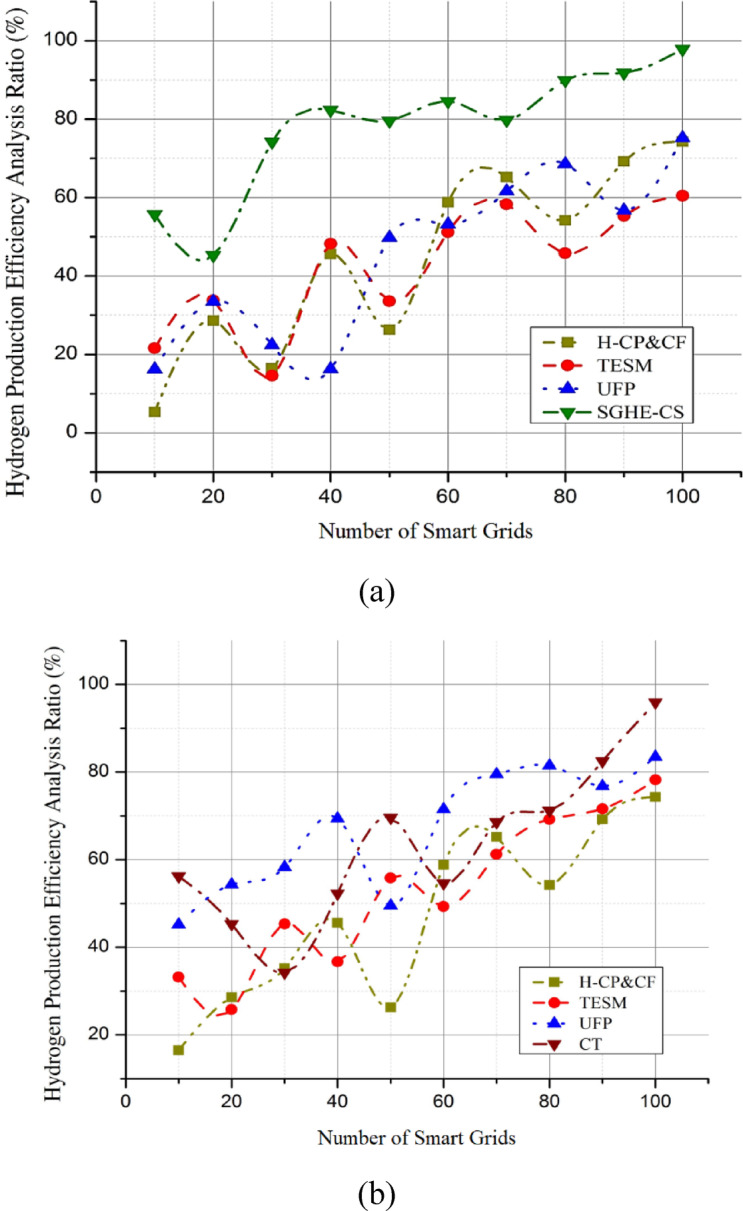
Hydrogen production efficiency compared with (**a**) SGHE-CS, (**b**) CT.

Improving hydrogen generation efficiency is crucial in discovering hydrogen’s potential and enhancing combustion wonders through smart grid interaction. Achieving maximum hydrogen production efficiency is crucial for the integrated system’s viability and sustainability. Identify whether the efficiency increases decreases, or remains constant over different conditions or periods for SGHE-CS and CT. Hydrogen production efficiency can be greatly improved using technologies like PEM electrolysis, significantly reducing energy losses during the electrolysis process. The suggested SGHE-CS uses state-of-the-art electrolysis technology to maximize renewable energy conversion into hydrogen. Efficient hydrogen production is crucial to align with smart grid dynamics and make real-time modifications for changing energy demands. The total effectiveness of renewable energy integration is strongly influenced by the methodical improvement of hydrogen production efficiency, stabilizing the grid. Efficient production of hydrogen, the backbone of the integrated system, is essential to fulfilling the potential of smart grid interface and enhanced combustion wonders, which in turn pave the path for a future energy that is both sustainable and low-carbon. Figure 6(a) shows that compared to the SGHE-CS, the hydrogen production efficiency reaches an impressive 98.5%. The improved performance of SGHE-CS in hydrogen production is highlighted by Fig. 6(b), which shows that CT attained an efficiency of 90.5%. Catalyst deterioration, membrane fouling, and electrolyzer material wear are the main causes of the progressive fluctuation in hydrogen generation efficiency over time. An increase in energy consumption per unit of hydrogen generated and a decrease in the rate of electrochemical reactions may occur when catalyst surfaces become inactive due to sintering, poisoning, or corrosion. Also, the total efficiency of the electrolyzer is diminished when contaminants, mechanical stress, or chemical assault produce membrane fouling or degradation, which in turn increases ionic resistance and gas crossover. Because these aging effects gradually lower hydrogen production efficiency during the operating lifetime, it is vital to undertake regular maintenance and replace components to maintain maximum performance.

### Storage and transportation

**Fig. 7:**
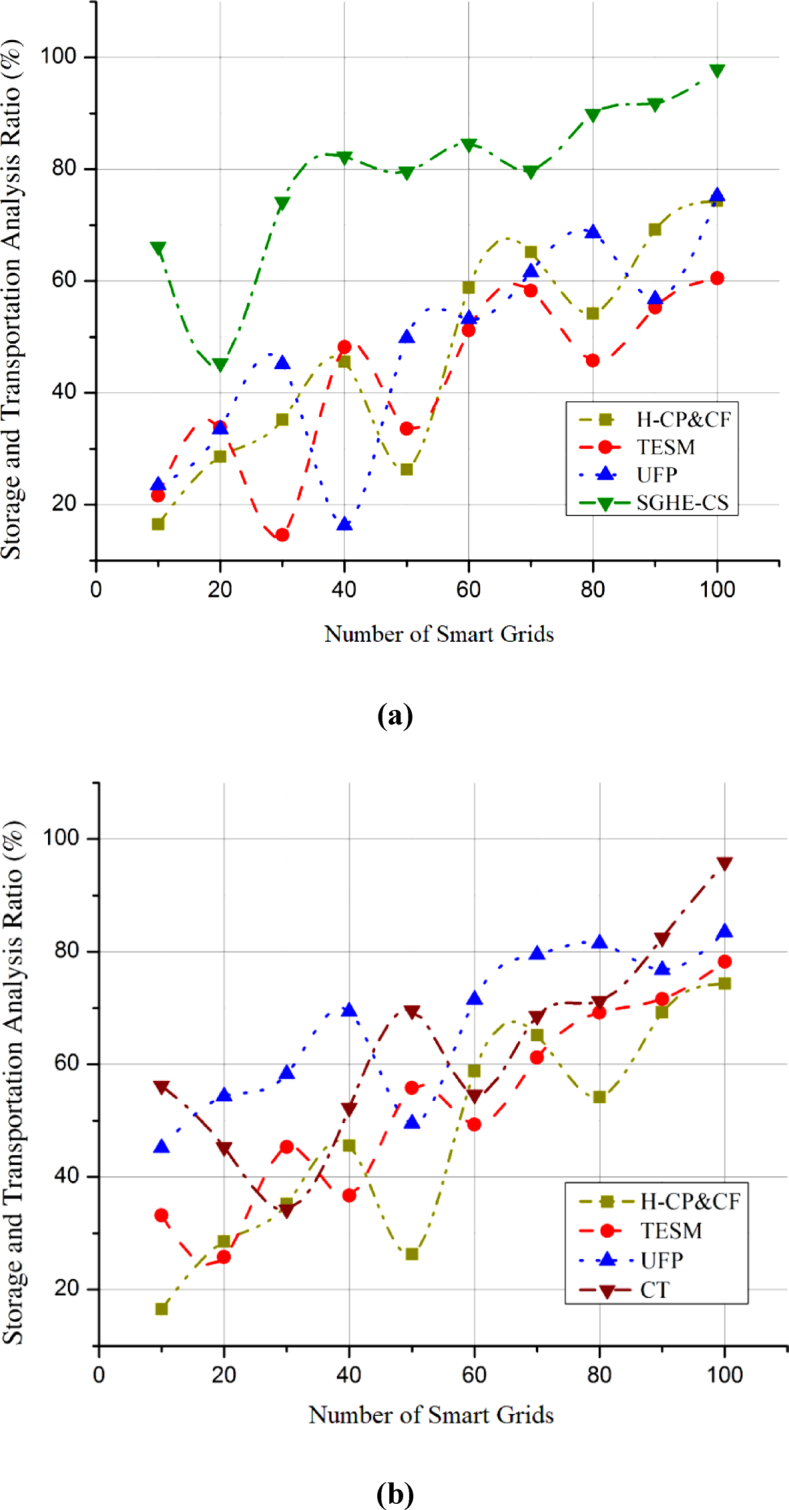
Hydrogen storage and transportation analysis (**a**) SGHE-CS, (**b**) CT.

Researching transportation and storage options is essential to revealing hydrogen’s possibilities and developing combustion wonders via smart grid interaction. Hydrogen storage efficiency is critical for a steady and dependable energy supply, especially in the ever-changing smart grid environment. To maximize reliability, safety, and flexibility, state-of-the-art solid-state hydrogen storage materials are crucial. The SGHE-CS may dynamically modify storage conditions through smart control systems using modern facilities storage technologies. Both storage and transportation curves are obtained by collecting data through datasets, plotting this data, and fitting curves to represent trends and efficiency. The key is to ensure that the curves accurately reflect the real-world performance of storage and transportation under varying conditions. This system can store and transport hydrogen efficiently by adjusting to different circumstances, reducing losses, and improving overall performance. A thorough examination of storage and transportation methods also helps with the problems of keeping the grid stable when demand is unpredictable; however, it makes incorporating hydrogen into many different kinds of applications easier. Prompting the development of clean energy solutions and adding to a sustainable and resilient energy future, the SGHE-CS optimizes transportation and storage through innovative approaches, becoming a crucial enabler in releasing hydrogen’s full potential within the complex framework of smart grid interaction. Compared to the SGHE-CS, the Storage and Transportation efficiency reaches 96.3% in Fig. 7(a). On the other hand, SGHE-CS excels in storage and transportation, as shown in Fig. 7(b), whereas CT only manages an efficiency of 89.1%. Storage and distribution of high-pressure compressed gas is this system’s main means of transporting hydrogen. The gas is usually kept in steel or composite cylinders at pressures ranging from 350 to 700 bar to make home-scale or local distribution uses of compressed hydrogen safe and efficient. The scattered nature of the smart grid system and infrastructural limitations make pipeline transport impractical for large-scale integration in this case. At the household scale, the high energy needs of liquefaction and boil-off losses make liquid hydrogen transport impracticable, so it is not used.

Compressed gas storage for hydrogen is the system’s main component, as it is the most common and viable technology for small-scale applications. A safe and efficient way to store compressed hydrogen is in high-pressure tanks, which can withstand up to 350 to 700 bar pressure. This makes for a space-saving storage option. Due to their greater prices, complexity, and extra safety or temperature control requirements, other techniques like liquid hydrogen or metal hydrides are less used at the home scale.

The storage of hydrogen must be done safely, particularly in homes. High-pressure tanks are built with state-of-the-art materials and safety features to provide maximum protection against rupture or leakage. These features include pressure release valves, impact resistance, and leak detection sensors. Further precautions must be taken to avoid the buildup of extremely combustible hydrogen gas, including installing hydrogen sensors and adequate ventilation. For household hydrogen storage systems to work safely, they must adhere to certain regulations and certifications throughout development, installation, and upkeep.

Considerations like available space, safety regulations, and the homeowner’s usual energy use should be considered when determining the hydrogen storage capacity. During low renewable output or grid failures, the storage system has to be big enough to provide energy but not so big that it causes needless expenses or space issues. To optimize storage capacity and provide a consistent, continuous power supply for homes, it is necessary to analyze daily and seasonal energy demand patterns, the unpredictability of renewable output, and the system’s efficiency in great detail.

### Integration with renewable energy sources analysis

**Fig. 8:**
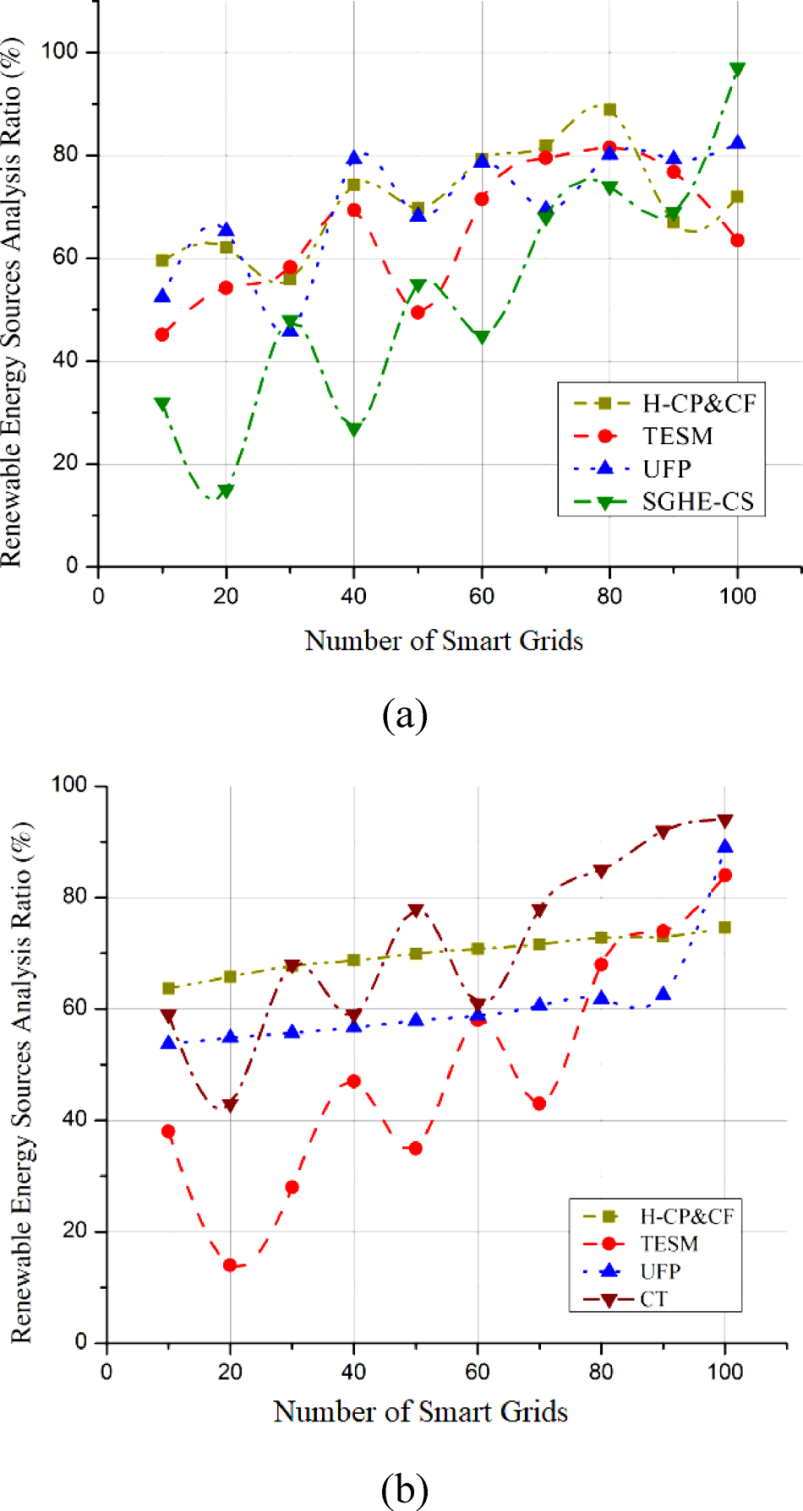
Integration with renewable energy sources analysis (**a**) SGHE-CS, (**b**) CT.

An in-depth investigation of hydrogen’s compatibility with renewable power sources is crucial to integrating its capabilities and technological advancements in combustion with a smart grid interface. The integration plays a crucial role in making the most of renewable energy by coordinating hydrogen production with the intermittent nature of renewable sources. The suggested SGHE-CS is engineered to coordinate hydrogen production with changes in renewable energy supply. The SGHE-CS helps create a greener energy system by using renewable energy better during high production times when prices are lowest. To ensure that hydrogen production can adjust to the changing energy scene, this analysis tackles problems caused by renewables’ transient nature. The smooth integration of hydrogen production with renewable energy sources improves grid stability and establishes hydrogen as a pivotal component in the shift towards greener energy. The seamless integration of hydrogen production with renewable energy sources enhances grid stability by balancing intermittent power generation and positioning hydrogen as a key element in the transition to sustainable energy. The analysis highlights the critical significance of smart grid interaction in integrating renewable sources with hydrogen technologies, creating a future with low-carbon energy, and strengthening the interrelated evolution of clean energy solutions through this integrated approach. Figure 8(a) shows that compared to the SGHE-CS, the integration with renewable energy sources analysis registers an impressive 97.3% efficiency. Figure 8(b) shows that CT achieves a respectable marginally lower efficiency of 92.3%. This exemplifies how well SGHE-CS works with renewable energy sources.

The high cost of electrolyzers, hydrogen storage, and combustion equipment is a major factor affecting the overall viability of the SGHE-CS system, which is a major obstacle to its implementation. Due to the intermittent nature of renewable inputs, complex control mechanisms must integrate with current grid infrastructure to preserve grid stability. Additionally, market preparedness is hindered by regulatory uncertainty related to hydrogen safety requirements, permits, and incentives. The optimization of system design for cost reduction, the enhancement of grid communication protocols, and the alignment with emerging legislative frameworks that favor hydrogen technologies and renewable integration are all necessary to address these difficulties.

### Combustion efficiency analysis

**Fig. 9:**
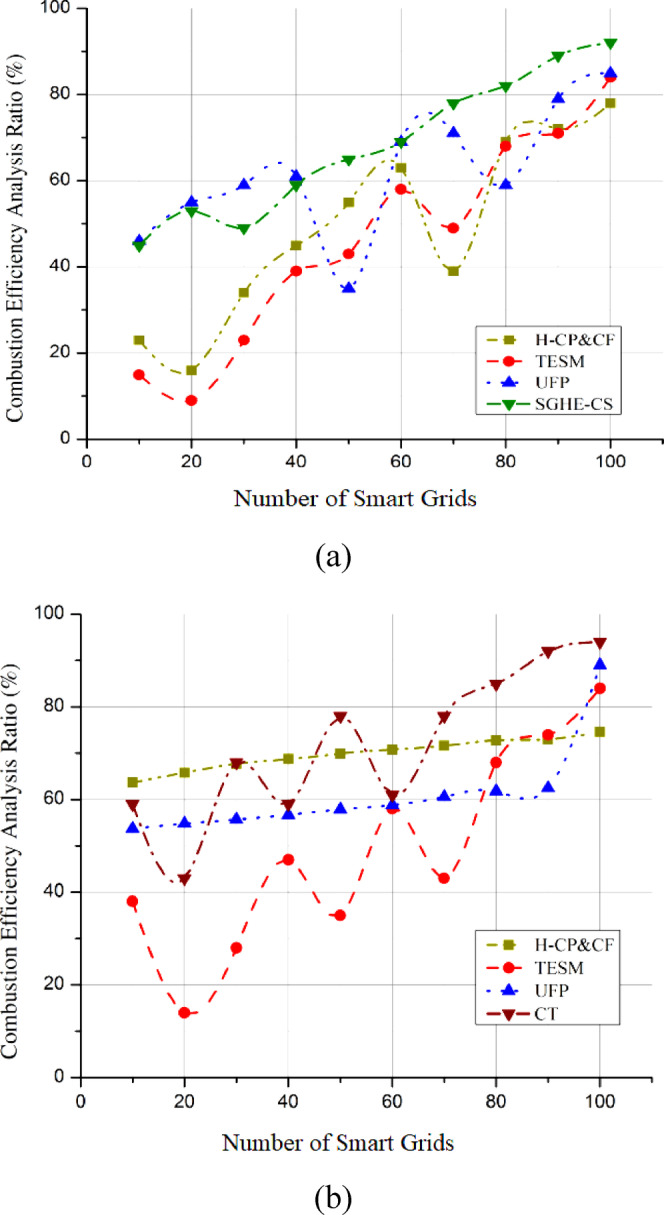
Combustion efficiency analysis (**a**) SGHE-CS, (**b**) CT.

Exploring the possibilities of hydrogen and developing combustion wonders through smart grid interaction necessitates comprehensively examining combustion efficiency. Improving combustion efficiency is crucial for getting the most energy out of hydrogen with the least environmental damage. The proposed SGHE-CS dramatically increases combustion efficiency using sophisticated catalytic and staged combustion approaches. These technologies ensure that hydrogen is used cleanly and completely to reduce pollutants and improve overall system efficiency. By integrating with smart grid interaction, dynamic modifications may be made to optimize the combustion process and match hydrogen combustion with real-time energy demands. Combustion efficiency curves are obtained by conducting experiments or simulations to gather data sets^26^ on how fuel is converted into energy. This data is then plotted to show efficiency trends under different conditions. Statistical or mathematical methods fit curves through the data, providing insights into how combustion efficiency varies with operational parameters. The combustion efficiency analysis lays the groundwork for cleaner, more sustainable energy practices and is crucial in resolving issues related to conventional combustion technologies. This research has two main contributions: first, it helps reduce carbon emissions by improving combustion efficiency within the context of smart grid interaction. Second, it positions hydrogen as an efficient and versatile energy carrier, crucial for transitioning to a low-carbon and environment-friendly energy future. Compared to the SGHE-CS, the combustion efficiency analysis displays a significant 98.1% efficiency in Fig. 9(a). On the other hand, CT attains an admirable relatively lesser efficiency of 92.4%, as shown in Fig. 9(b). This exemplifies SGHE-CS’s notable combustion efficiency.

### Versatility analysis

**Fig. 10:**
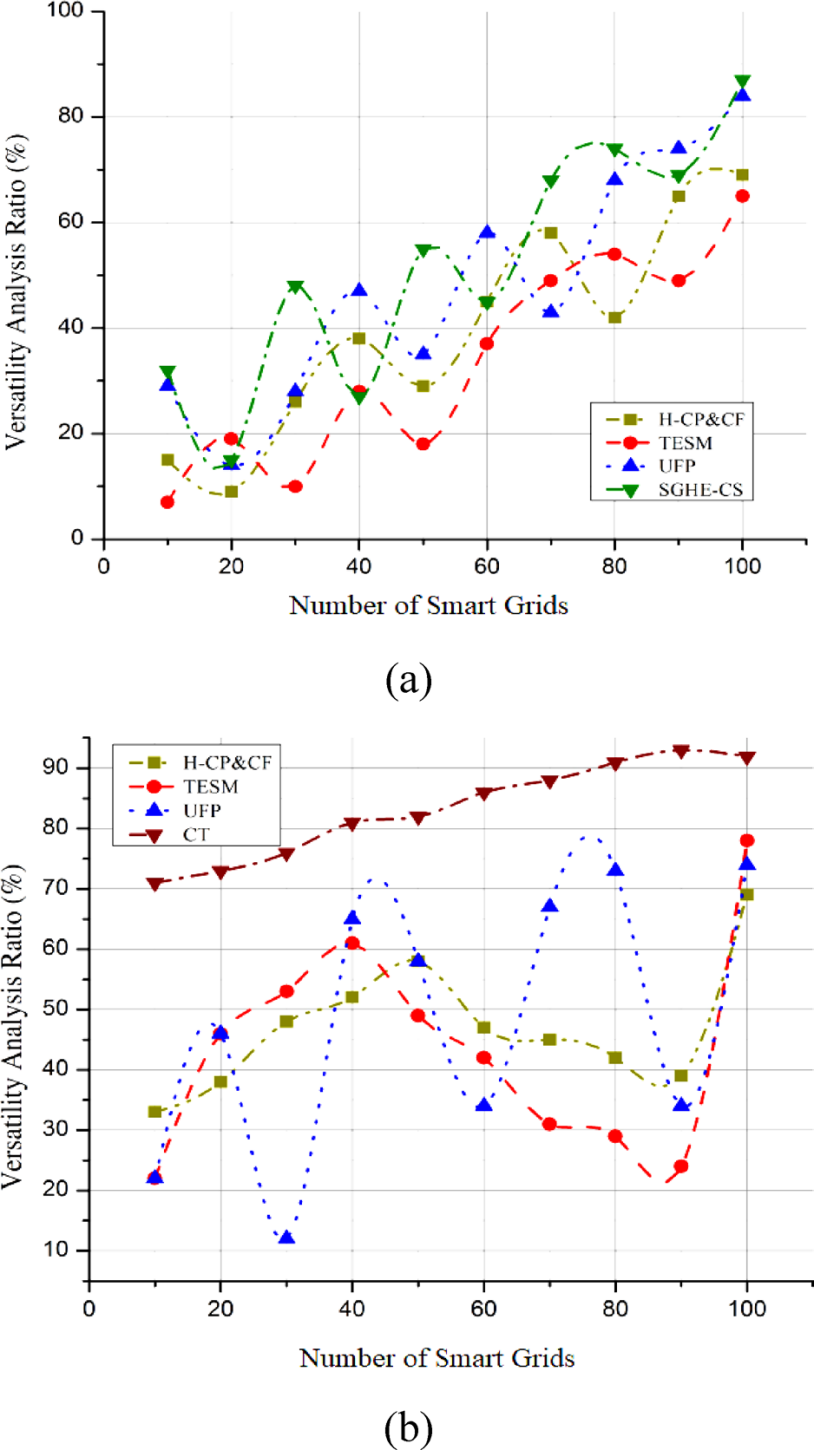
Versatility analysis ratio (**a**) SGHE-CS, (**b**) CT.

The effectiveness of integrated clean energy systems depends on a thorough investigation of hydrogen’s adaptability in proving its potential and improving combustion performance by employing smart grid integration. Assessing or simulating the system across various conditions or inputs helps one develop versatility analysis curves, therefore assessing its adaptability and performance. Graphically displayed data from these tests or simulations shows performance or efficiency variances concerning different criteria. Curves are adapted to the data using statistical or mathematical methods, clarifying the system’s adaptability. The proposed SGHE-CS aims to show the adaptability of hydrogen as an energy carrier with numerous conceivable uses. Integration of hydrogen across many sectors, including transportation, manufacturing, and power generation, emphasizes its vital importance in solving the challenges presented by different energy demands. Because of its link with the smart grid, which allows real-time changes, the SGHE-CS can efficiently respond to changing energy demands. The flexibility study guarantees its utility throughout many situations by evaluating the efficiency of hydrogen generation and combustion within a dynamic energy environment. This paper investigates the possibilities of hydrogen as a flexible, robust, environmentally friendly energy source employing analyses of its manufacturing and combustion techniques. When smart grid interaction and hydrogen’s adaptability are combined, a cleaner, more adaptable, and economically feasible energy future is within reach. Figure 10(a) shows that compared to the SGHE-CS, the Versatility Analysis demonstrates an exceptional efficiency of 99.3%. However, CT manages a 90.5% versatility efficiency, as shown in Fig. 10(b). This highlights how SGHE-CS outperforms more conventional options in terms of its adaptability. The fundamental Equation for grid stability revolves around maintaining the balance between power generation and consumption. $\:{P}_{gen}={P}_{load}+{P}_{loss}$​.

Ultimately, the SGHE-CS proves to be a game-changing technology, continually surpassing traditional alternatives in terms of efficiency across a range of measures. Sustainable and efficient energy systems of the future may be shaped by SGHE-CS, recognition of its versatility, integration of sophisticated technology, and real-time changes. Complex grid stability management is necessary for large-scale renewable energy integration. Blackouts, voltage instability, and frequency aberrations are all possible outcomes of power production fluctuations that cannot be corrected in real time. The dependent variables’ values (such as the rate of hydrogen synthesis, storage efficiency, and combustion output) exhibit oscillatory behavior because of the dynamic interplay between renewable energy inputs and system balancing mechanisms. As more smart grids are introduced, the unpredictability of solar and wind energy inputs leads to temporal changes in generation and demand, directly affecting the hydrogen production and storage cycles. A system with real-time feedback and adaptive control, like SGHE-CS, exhibits these dynamics to a greater extent.

Evaluating the feasibility of hydrogen-based energy systems requires considering not just emissions and efficiency but also the cost of generation and storage of hydrogen. The primary factors impacting production costs are the initial investment in electrolyzers, ongoing operating expenditures like power input (particularly when generated from renewable sources), and maintenance costs associated with defective components. A key factor influencing total production costs is the efficiency of the electrolyzer, which in turn impacts the amount of energy used per kilogram of hydrogen. Regarding storage, the costs can vary greatly depending on the technology used. For example, high-pressure vessels with strong safety features are needed for compressed gas storage, cryogenic infrastructure with a lot of energy input for liquefaction is necessary for liquid hydrogen, and advanced options, such as metal hydrides, can involve expensive materials and complicated thermal management. The optimal levelized cost of hydrogen (LCOH) is achieved when system efficiency is considered alongside these capital and operating expenses. In addition, the system’s economic viability and sustainability are heavily influenced by emissions factors, such as indirect emissions from power generation and the lifecycle effects of storage solutions.

The efficiency of the SGHE-CS system was tested under several input situations, such as a variable renewable power source and partial load operation, using a sensitivity analysis. At 40% load, the electrolyzer’s efficiency dropped from 78% at rated capacity to around 70%, which was caused by poorer catalyst utilization and greater relative ohmic losses. Similarly, since the turbine’s intake temperatures and pressure ratios weren’t ideal, the combustion turbine’s efficiency dropped from 90.5% full load to over 82% 50% load. Transient variations in electrolyzer ramp rates and gas turbine load following caused the total system efficiency to vary between 85% and 93% when modeling intermittent solar and wind inputs with variability factors up to 30%.

## Conclusions

A sustainable and low-carbon energy future is within reach, especially in the presented investigation highlighting the critical role of smart grid interaction in developing combustion wonders and maximizing hydrogen’s potential. Intending to optimize the smart grid and coordinate hydrogen production with fluctuating energy demands, the study recommends a holistic approach. Promising a real-time data interchange to synchronize hydrogen production with changes in renewable energy supply, the suggested SGHE-CS is a viable alternative. Maximizing energy extraction while minimizing emissions from hydrogen combustion is the goal of incorporating staged combustion techniques and advanced catalysts. In addition to overcoming current limitations and broadening the achievable boundaries, the research includes breakthroughs in hydrogen generation techniques, storage technologies, and combustion methodologies. Efficient and flexible system performance evaluation in real-time is made possible using cutting-edge monitoring technologies. Demonstrating the method’s applicability requires the validation of the SGHE-CS through simulation analyses. These analyses should evaluate its efficiency, environmental impact, and adaptability across numerous applications. By successfully tackling issues with hydrogen consumption and utilizing the capabilities of the SGHE-CS, this research aims to illuminate the revolutionary potential of smart grid technology, which entails a more adaptive and greener energy landscape. The experimental outcomes demonstrate that the versatility analysis of 99.3%, combustion efficiency of 98.1%, renewable energy integration of 97.3%, storage and transportation efficiency of 96.3%, and hydrogen production efficiency of 98.5% are achieved compared to other existing techniques.

## Data Availability

The data that supports the findings of this study are available in the article and can be obtained from the Corresponding author on reasonable request.

## Abbreviations

SGHE-CS: Smart grid hybrid electrolysis-and combustion system
UFP: Uspekhi forum presentation
PEM: Proton exchange membrane
MRs: Microreactors
SMRs: Small modular reactors
AA-RMD: Algorithmic approaches for rational material design
AC: Activated carbon
$\:{\omega\:I2}_{Prod}$: Total efficiency of hydrogen production
$$\:{\theta\:}_{{I2}_{j}\left(u\right)+1}$$: Dynamic parameters
$\:{\delta\:}_{{I2}_{j}}$: Logarithmic function-related constants
$\:{x}_{{I2}_{j}}$: Function-associated frequencies
$\:{u}_{empty}$: Replenish the hydrogen storage empty
$$\:\left(1-{f}^{-\frac{{u}_{storage}}{{\tau\:}_{decay}}}\right)$$: Decay factor
$\:{u}_{storage}$: Total storage period
$\:{\tau\:}_{deacy\:}$: Time taken for the decay constant
$\:{\omega\:}_{transport}$: Combination of transportation
$$\:{\omega\:}_{storage}$$: Efficiency factors
$\:{N}_{I2}$: The molecular weight of hydrogen
$\:{Q}_{renewable}$: Renewable sources production
$$\:{Q}_{ambient}$$: Ambient strength
$\:{Q}_{demand}$: Regard to time
$$\:HCV$$: Heating value
$\:{\omega\:}_{complete}$: Total combustion efficiency
$$\:{n}_{fuel}$$: Fuel mass
$\:{\overrightarrow{F}}_{renewable}$: Electrical field vector
$\:{\overrightarrow{Q}}_{demand}$: Power demanding vector
$\:\gamma\:$: The magnetic induction efficiency
$\:{D}_{emission}$: The combustion efficiency
$\:{Q}_{P2}$: Oxygen partial pressure
$\:{Q}_{I2}$: Hydrogen partial pressure
